# Exosomal microRNAs from Alveolar Macrophages Reveal a Protective Role of the Lung Microbiome Against Oncogenic Signaling During PAH Exposure

**DOI:** 10.3390/cells15080715

**Published:** 2026-04-18

**Authors:** Harish Chandra, Brijesh Yadav, Damaris Kuhnell, Scott Langevin, Jacek Biesiada, Mario Medvedovic, Jagjit S. Yadav

**Affiliations:** 1 Pulmonary Pathogenesis and Immunotoxicology Laboratory, Department of Environmental and Public Health Sciences, College of Medicine, University of Cincinnati, Cincinnati, OH 45267, USA; 2 Division of Epidemiology, Department of Environmental and Public Health Sciences, College of Medicine, University of Cincinnati, Cincinnati, OH 45267, USA; 3 Division of Biostatistics and Bioinformatics, Department of Environmental & Public Health Sciences, College of Medicine, University of Cincinnati, Cincinnati, OH 45267, USA

**Keywords:** exosomes, extracellular vesicles, alveolar macrophages, lung microbiome, aryl hydrocarbon receptor, polycyclic aromatic hydrocarbons

## Abstract

Polycyclic aromatic hydrocarbons (PAHs), such as benzo[a]pyrene (B[a]P), are major risk factors for lung cancer and other diseases, acting through the aryl hydrocarbon receptor (AHR). Alveolar macrophages (AMs) help regulate the lung microenvironment by responding to inhaled toxicants and resident microbiota. Although small extracellular vesicles (sEVs, aka exosomes) released by AMs mediate intercellular communication and immune responses, the influence of lung microbiota on sEV biogenesis and the mechanisms underlying sEV dysregulation during PAH exposure remain unknown. Here, we investigated the interplay between AMs, B[a]P, and lung microbiota, focusing on sEV-associated miRNAs (exo-miRNAs). Murine AMs (MH-S) were exposed to varying B[a]P concentrations in the presence or absence of murine lung microbiota with or without an AHR antagonist. sEVs from each condition were characterized and profiled for miRNA. Distinct miRNA signatures emerged: high-dose B[a]P enriched miRNAs linked to cancer progression, whereas lung microbiota alone or with low-dose B[a]P induced tumor-suppressor miRNAs that limit proliferation and metastasis and promote apoptosis, an effect enhanced by AHR antagonism. Lung microbiota appeared to counteract high-dose B[a]P by modulating tumor-suppressive exo-miRNAs. This study demonstrates that lung microbiota-induced exo-miRNAs critically shape AM-derived sEV-miRNA signaling during PAH exposure. The identified exosomal miRNAs could serve as important exposure biomarkers and therapeutic targets for mitigating B[a]P-induced toxicity and cancer development.

## 1. Introduction

Human lungs are frequently exposed to polycyclic aromatic hydrocarbons (PAHs), including carcinogenic members such as benzo[a]pyrene (B[a]P), via smoking and air pollution from traffic emissions, fuel/biomass burning, and other air-borne hazards, increasing the risk of cancer and other respiratory diseases [1,2]. Inhaled PAHs impact resident immune cells of the respiratory tract, including alveolar macrophages, which shape innate and adaptive immunity [3,4]. Traditionally, the lungs have been considered a sterile organ, but modern culture-independent next-gen sequencing approaches for microbial analysis have unambiguously demonstrated that lungs harbor a dynamic commensal microbiome [5,6]. Therefore, a dynamic interaction between the lung immune system, especially alveolar macrophages, and the lung microbiota takes place, modulating lung immunity [6,7,8,9]. In the event of exposure to PAHs, which are known to dampen the immune system, this intricate balance might get compromised, resulting in disruption of homeostasis and disease manifestation. However, the nature of such an impact and the underlying mechanisms during exposure to PAHs in the lungs are unclear.

There has been considerable recent interest in small extracellular vesicles (“exosomes”), which are released from all eukaryotic cells, including immune cells, and are involved in a variety of immune functions [10]. Exosomes are small nano-sized vesicles (40–150 nm diameter) that are made up of lipid bilayer membranes enclosing biologically active mRNAs, proteins, lipids, and microRNA (miRNA) species from the source cells and have been shown to participate in immune responses by interacting with different target cells [10,11]. Small extracellular vesicles (sEVs; ≤200 nm diameter), which include both exosomes and small microvesicles, can be recovered from essentially all biofluids, including saliva, lung lavage, serum, urine, as well as conditioned cell culture media [10]. Increasing evidence suggests that sEVs function as carriers of biologically active genetic information and play an active role in cell–cell communication [12]. sEVs released by alveolar macrophages carry molecular cargo that may regulate multiple immunological processes [13,14]. On the other hand, exposures to PAHs have been shown to alter miRNA expression in cells that play an important role in cancer progression and development via negative regulation of genes in associated pathways [15].

Growing evidence indicates that exosomal miRNAs represent a critical layer of post-transcriptional regulation during environmental exposures and immune responses. Because miRNAs directly target mRNAs encoding key regulatory proteins involved in inflammation, xenobiotic metabolism, epithelial integrity, and carcinogenic signaling, changes in their abundance can profoundly influence cellular behavior. Exosomal miRNAs are particularly important because they are selectively packaged, stable in circulation, and capable of modulating gene expression in recipient cells, making them powerful mediators of intercellular communication. These properties also position exosomal miRNAs as promising biomarkers of toxicant exposure and early disease processes, as well as potential therapeutic targets for modulating dysregulated pathways in respiratory disorders. However, how PAH exposure and the lung microbiome jointly shape the exosomal miRNA landscape of alveolar macrophages remains unknown.

In view of the above scientific premise, one may therefore rationalize that exosomes released from the PAH-exposed immune cells might carry important miRNA species relevant to associated disease conditions of the respiratory tract. However, nothing is known regarding how PAH exposure alters exosomes in naïve and microbiota-sensitized immune cells. We postulate that environmental exposure to PAHs alters the homeostatic interaction between lung immune cells and the resident lung microbiome in terms of exosomal miRNA profile, triggering biological responses underlying inflammatory/carcinogenic conditions of the respiratory tract. Characterization of the altered sEV miRNAome might reveal important biological markers or gene networks impacted by the interaction of inhaled PAHs, immune cells, and the lung commensal microbiota. Therefore, this current study investigates the effect of the prototype carcinogenic environmental PAH (B[a]P) on resident lung microbiota interactions with alveolar macrophages (AMs) in terms of release of sEVs and their miRNA cargo (exo-miRNA) using comprehensive miRNA-sequencing (miRNA-seq) and target genes/pathways analyses. This study provided hitherto unknown evidence on the role of the lung commensal microbiome in inducing sEVs from alveolar macrophages. Molecular characterization revealed differential miRNA signatures in the AM-released sEVs during interaction with resident lung microbiota versus inhaled PAH, implying a protective role of the lung microbiome in PAH-induced toxicity and carcinogenesis.

## 2. Materials and Methods

### 2.1. Isolation of Mouse Lung Microbiota

All animal experiments were done according to the University of Cincinnati’s Institutional Animal Care and Use Committee (IACUC)-approved protocol. Healthy inbred C57BL/6 adult female mice (8–10 weeks old) purchased from Jackson Laboratory (600 Main Street, Bar Harbor, ME 04609, USA) were acclimatized (one week) and housed in the University of Cincinnati’s pathogen-free Laboratory Animal Medical Services (LAMS) facility before isolation of resident lung microbiota. For this, mouse lungs were lavaged with phosphate-buffered saline (1× PBS) according to our established protocol [6]. The bronchoalveolar lavage (BAL) fluid was centrifuged at 4 °C in two steps to differentially separate lung cells (1000 RPM for 5 min) first, followed by pelleting of microbial cells (12,000 rpm for 5 min). The microbiota pellets from individual mice BAL fluids were pooled from a group of four mice and resuspended in a total volume of 1 mL.

### 2.2. Alveolar Macrophage (AM)-Culturing and Treatment Groups

Murine alveolar macrophage cell line MH-S (CRL-2019), purchased from American Type Culture Collection (ATCC), (Manassas, VA, USA) and maintained as described elsewhere [16], was used in this study as a source of cultured alveolar macrophages (AMs). For exosome release experiments, MH-S cells grown to confluency in T175 flasks were washed and resuspended (10 × 10^6^ cells/flask) in fresh RPMI medium containing 10% sEV-depleted fetal bovine serum (FBS). Using same-size culture T-flasks, four replicates of MH-S cell suspensions were prepared and treated, followed by incubation for 36 h. For microbiome (Mb) treatment experiments, an aliquot of the isolated microbiota pool from the lungs of the mice was used to treat the MH-S cells. The following experimental groups were generated by treating with B[a]P (low concentration (1 µg/mL) versus high concentration (10 µg/mL)) and/or the microbiome (a 30 µL aliquot of the isolated lung microbiome pool) as follows: (1) vehicle-only treated cells (DMSO treatment to a final concentration of 0.03% served as the vehicle control); (2) microbiome-only treated cells (30 µL microbiota suspension); (3) B[a]P-only treated cells (low concentration: 1 µg/mL); (4) B[a]P-only treated cells (high concentration: 10 µg/mL); (5) microbiome (30 µL) +B[a]P (1 µg/mL) treated cells; (6) microbiome (30 µL) +B[a]P (10 µg/mL) treated cells; (7) microbiome (30 µL) + B[a]P (1 µg/mL) treated cells in the presence of 5 µM AHR antagonist CH223191 (Cayman Chemical; Ann Arbor, MI, USA); and (8) microbiome (30 µL) + B[a]P (10 µg/mL) in the presence of 5 µM AHR antagonist CH223191. Cell-free supernatants (conditioned media) were collected from individual treatment groups, aliquoted into 15 mL conical tubes, and frozen at −80 °C for subsequent experiments.

### 2.3. Small Extracellular Vesicle Isolation

sEVs were isolated from 30 mL of conditioned media, by being subjected to a series of differential centrifugation cycles to remove cellular debris, each time retaining the supernatant (300× *g* for 5 min > 1200× *g* for 20 min > 10,000× *g* for 30 min). The supernatant was then centrifuged at 100,000× *g* for 70 min in an L8-60M ultracentrifuge (Beckman Coulter, Brea, CA, USA) using a 70Ti fixed-angle rotor. The resulting supernatant was discarded, and the pellet was resuspended in 500 µL PBS buffer. sEVs were isolated from the resuspension using the MagCapture Exosome Isolation Kit PS (Wako Chemicals, Osaka, Japan) according to the manufacturer’s protocol.

### 2.4. sEV Characterization

The size range and concentration for each sEV isolation was determined via nanoparticle tracking analysis (NTA) using a NanoSight NS300 instrument (Malvern, Worcestershire, UK), based on five replicates for each sample. sEV isolates were diluted 1:100 in PBS, with the instrument set to camera level 14 and detection threshold = 5.

sEVs were visually confirmed by transmission electron microscopy (TEM) imaging with a JEOL JEM-1230 instrument (JEOL USA, Inc., Peabody, MA, USA). To prepare the sample, a drop of 0.1% bovine serum albumin (BSA) was placed on a formvar carbon-coated grid for 1 min and then wicked away with a piece of filter paper. We then placed 10 µL of the sEV isolate on the grid for 5 min, followed by gently wicking the sample away and adding 10 drops of 2% aqueous uranyl acetate (UA) to the grid. The UA was then wicked away and the grid allowed to dry before imaging.

The presence of sEVs was further confirmed by Western blot analysis for the EV-associated tetraspanin CD9 and cytosolic endosomal sorting complex component TSG101 [17]. Western blots were run using NuPAGE 4–12% Bis-Tris Gels (Thermo Fisher Scientific, Waltham, MA, USA) in a mini gel tank with MOPS SDS Running Buffer with added NuPAGE Antioxidant. A 30 µL aliquot of sEV isolate was loaded for each of the samples and for the negative control; 5 µL of sEV isolate was loaded from the positive control (Detroit 562 HNSCC cell line). Samples were mixed with 4× Laemmli SDS sample buffer (non-reducing) and NuPAGE Reducing Agent (10×) and heated to 95 °C for 7 min. The gel was run for 50 min at 200 V constant. Proteins were subsequently transferred onto a PVDF membrane, using Pierce™ 1-Step Transfer Buffer on a Pierce Power Station (Thermo Fisher Scientific, Waltham, MA, USA) for 10 min at 1.3 A constant. CD9 (SBI:EXOAB-CD9A-1, System Biosciences, Palo Alto, CA, USA) antibody and TSG101 (ab30871, Abcam, Cambridge, UK) antibody were added at 1:1000 dilution in 5% Milk and 5% BSA, respectively, in Tris buffer saline with 0.1% tween 20 (TBST) overnight at 4 °C. After washing the membrane for 3 × 5 min in TBST, secondary antibody Goat anti-Mouse IgG H&L (ab205719, Abcam) was added at 1:3000 in 5% Milk in TBST for three hours for CD9, and secondary antibody Goat anti-Rabbit IgG H&L (ab205718, Abcam) was added at 1:2000 in 5% BSA in TBST for two hours for TSG101. Following three final washes at 5 min each, detection was performed using a Western Bright ECL detection kit (Advansta, Menlo Park, CA, USA) on a C-DiGit Blot Scanner (LI-COR Biotechnology, Lincoln, NE, USA).

### 2.5. Total RNA Isolation and miRNA Sequencing

Total small RNA (≤200 bp) fraction was isolated from sEV isolates using the MirVana miRNA isolation kit (Invitrogen, Carlsbad, CA, USA) according to the manufacturer’s protocol for total RNA isolation. Briefly, sEVs were lysed with exosome lysis buffer and mixed with miRNA homogenate additive, followed by mixing with acid phenol chloroform and RNA precipitation with absolute ethanol. The miRNA library preparations and sequencing were performed by the University of Cincinnati’s Genomics, Epigenomics and Sequencing Core. To prepare the library, the NEBNext small RNA sample library preparation kit (New England BioLabs, Ipswich, MA, USA) was used with ~5 ng of total RNA determined by the Bioanalyzer RNA 6000 Pico Kit (Agilent, Santa Clara, CA, USA) in a 5 µL solution as input, following the manufacturer’s protocol except for a modification of library size selection to increase small RNA detection sensitivity and specificity. After 15 cycles of PCR for indexing and library enrichment, an equal volume of 10 μL PCR mix (library without size selection) per sample together with the same volume of the negative control were pooled, followed by DNA cleanup using DNA Clean & Concentrator (Zymo Research, Irvine, CA, USA) and mixed with 135 and 319 bp custom-made ladders targeting the library’s 16–24 miRNA cDNA insert. Next, precise size selection of the 135–319 bp library via 2.75% agarose gel electrophoresis was performed, and library concentration was measured by qPCR using the NEBNext Library Quant Kit (New England BioLabs) on a QuantStudio 5 Real-Time PCR System (Thermo Fisher Scientific). Quantified libraries were clustered onto a flow cell at a concentration of 15 pM using the TruSeq SR Cluster Kit v3 (Illumina, San Diego, CA, USA) and sequenced for 51 cycles using TruSeq SBS kit on a HiSeq 1000 system (Illumina) to generate a few million reads. Based on the sequencing read number from each sample, an equal-read number pool from the PCR mix was calculated via volume adjustment of the PCR mix. Finally, the same procedure for the second round of sequencing was performed to generate the expected number of reads for final data analysis.

### 2.6. Bioinformatic and Statistical Analyses

We performed all experiments in triplicate. Differential expression of genes and target enrichment were analyzed using the Statistical and genomics core facility services of the University of Cincinnati. For bioinformatics analysis, the data was first cleaned by removal of adaptors and bad reads like NNNNNNN, followed by performance of quality control (QC); thereafter, the reads were aligned and the counts were quantified. Differential expression on assigned groups was performed, and prediction of targets and enrichment analyses were done. All analysis was done in R (https://www.r-project.org/) with packages designed for microRNA-seq targets and enrichment. We used the mm10 reference genome for alignment using the aligner Bowtie (version 0.12.7) (http://bowtie-bio.sourceforge.net/index.shtml, accessed on 8 November 2018). All counts were quantified with the count Overlaps function from the Genomic Features (Bioconductor) package (https://www.bioconductor.org/), based on the mirBase definition of microRNA genes http://www.mirbase.org/.

Quality control assessment was done by FastQC correlation plot for samples (https://www.bioinformatics.babraham.ac.uk/projects/fastqc/, accessed on 8 November 2018). Differential miRNA analysis was performed using the edgeR package (Version 3) (https://bioconductor.org/packages/release/bioc/html/edgeR.html, accessed on 8 November 2018). For miRNA target pathway enrichment, multiMiR was used for finding validated miRNA targets (https://bioconductor.org/packages/release/bioc/html/multiMiR.html, accessed on 8 November 2018). A collection of miRNAs/targets from external resources was used, including validated miRNA-target databases (miRecords, miRTarBase, and TarBase), predicted miRNA-target databases (DIANA-microT, ElMMo, MicroCosm, miRanda, miRDB, PicTar, PITA, and TargetScan) and miRNA-disease/drug databases (miR2Disease, Pharmaco-miR VerSe, and PhenomiR). Enrichment analysis was performed with the CLEAN package (http://eh3.uc.edu/clean/, accessed on 8 November 2018). The statistical significance of differential expression was established based on the FDR-adjusted *p*-values.

KEGG pathway enrichment analysis was performed with DIANA mirPath v.3. as annotated in DIANA-TarBase v.7.0. miRNA target gene interaction maps were generated using the miRNet platform (https://www.mirnet.ca/, accessed on 8 November 2018). Significantly enriched functional categories were identified using Fisher’s test.

## 3. Results

### 3.1. Detection and Characterization of sEVs (Exosomes) in Lung Macrophage–Toxicant–Microbiome Interactions

**Figure 1:**
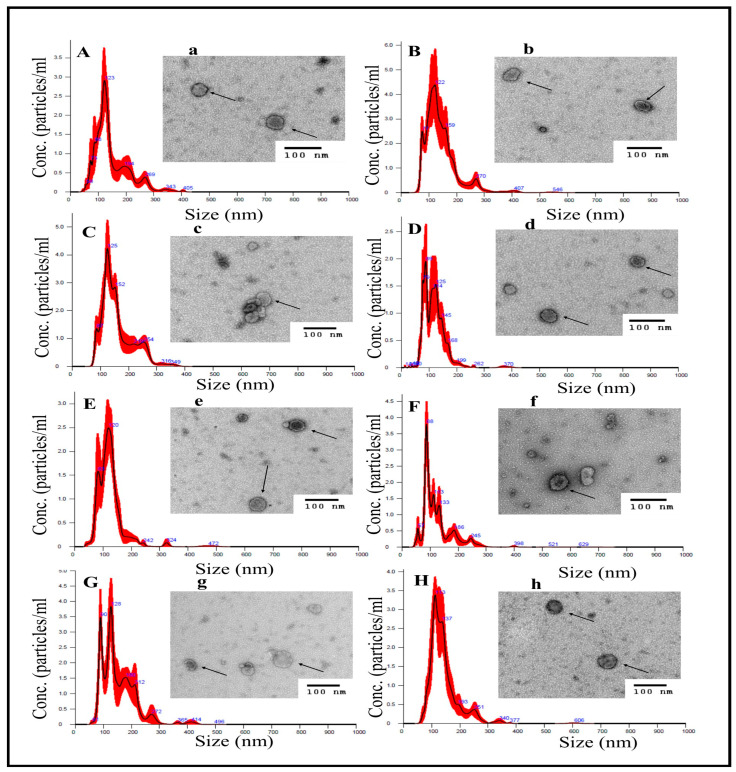
Characterization of small extracellular vesicles (sEV) isolated from variously treated cultured murine alveolar macrophages (MH-S cell line). **Left** panels (**A**–**H**): Size distribution plots from nanoparticle tracking analysis according to particle diameter. (**A**) Vehicle-only treated MH-S cells. (**B**) Microbiome-only treated MH-S. (**C**) B[a]P-only treated MH-S (low concentration; 1 μg/mL). (**D**) B[a]P-only treated MH-S (high concentration; 10 μg/mL). (**E**) Microbiome + B[a]P (1 μg/mL). (**F**) Microbiome + B[a]P (10 μg/mL). (**G**) Microbiome + B[a]P (1 μg/mL) in the presence of AHR antagonist CH223191. (**H**) Microbiome + B[a]P (10 μg/mL) in the presence of AHR antagonist CH223191. **Right** panels (**a**–**h**) Representative transmission electron microscopy (TEM; 200,000×) images of sEVs isolated from each respective treatment group are presented in the right panel of each plot; arrows highlight representative sEVs. A 100 nm scale bar is provided in the bottom right-hand corner of each image for perspective. Abbreviations; Conc. (concentration).

The particle concentration and size distribution of the sEV isolates, along with representative TEM images, are depicted in Figure 1. All treated groups showed similar profiles in terms of size, shape, and concentrations of these vesicles (Figure 1 and Supplementary Table S1). The size distribution by NTA showed an average particle size < 150 nm.

**Figure 2:**
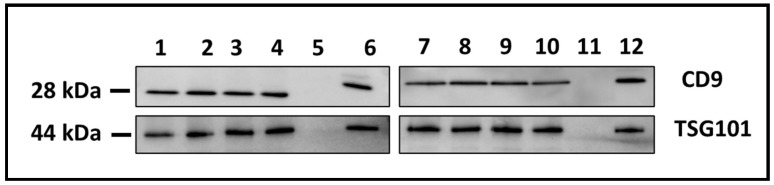
Expression of the extracellular vesicle protein markers CD9 (top panel) and TSG101 (bottom panel) in small extracellular vesicles (sEVs) isolated from the culture medium of different treatment groups. Lane 1: Vehicle-only treated MH-S cells. Lane 2: Microbiome-only treated MH-S. Lane 3: B[a]P-only treated MH-S (low concentration; 1 μg/mL). Lane 4: B[a]P-only treated MH-S (high concentration; 10 μg/mL). Lane 5: Negative control (PBS only). Lane 6: Positive control. Lane 7: Microbiome + B[a]P (1 μg/mL). Lane 8: Microbiome + B[a]P (10 μg/mL). Lane 9: Microbiome + B[a]P (1 μg/mL) in the presence of AHR antagonist CH223191. Lane 10: Microbiome + B[a]P (10 μg/mL) in the presence of AHR antagonist CH223191. Lane 11: Negative control (PBS only). Lane 12: Positive control.

The presence of sEVs was further confirmed based on the expression of hallmark biological markers of EVs, namely membrane tetraspanin CD9 and the cytosolic endosomal sorting complex component TSG101 by Western blotting [18]. All treatment groups expressed both markers with comparable intensity (Figure 2).

### 3.2. Differential Expression of Significant miRNAs in sEVs

**Figure 3:**
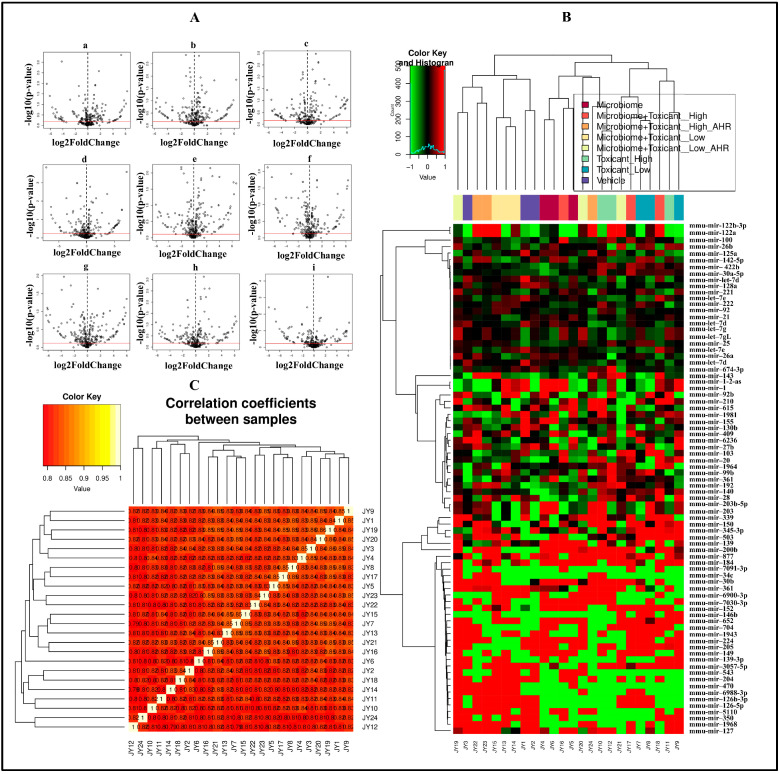
Differential expression patterns of miRNA in small extracellular vesicles (exo-miRNAs) from cultured alveolar macrophages across different treatment groups. Volcano plots (**A**): Microbiome vs. Vehicle (**a**); Low Toxicant vs. Vehicle (**b**); High Toxicant vs. Vehicle (**c**); Microbiome + Low Toxicant vs. Microbiome (**d**); Microbiome + Low Toxicant + AHR vs. Microbiome + Low Toxicant (**e**); Microbiome + Low Toxicant vs. Low Toxicant (**f**); Microbiome + High Toxicant vs. High Toxicant (**g**); Microbiome + High Toxicant vs. Microbiome (**h**); Microbiome + High Toxicant + AHR vs. Microbiome + High Toxicant (**i**). Heat map of differential exo-miRNA expression (**B**) and correlation coefficients (**C**) between samples for differentially expressed exo-miRNA (though this software auto-generated figure has an unavoidable overlap between the third digit and the second digit after the decimal, the first digit shows a high correlation value (approximately 0.8) for all samples). The horizontal red line in each volcano plot corresponds to *p* = 0.05, adjusted for false discovery rate (FDR). Black vertical dashed lines in the volcano plots divide specific exo-miRNA transcripts—black dots to the right correspond to an upregulation, and those to the left correspond to a downregulation in secreted level. Sample ID (JY1–JY24) information is described separately in the Supplementary Materials. The label Toxicant represents B[a}P, with Low (1 µg/mL) and High (10 µg/mL) representing the two concentrations used. The label AHR here represents the AHR antagonist.

Differential expression patterns of significant exo-miRNAs were analyzed across various treatment groups using heat maps and volcano plots (Figure 3). These differential expression patterns have been described in the following sections under different treatment groups.

**Figure 4:**
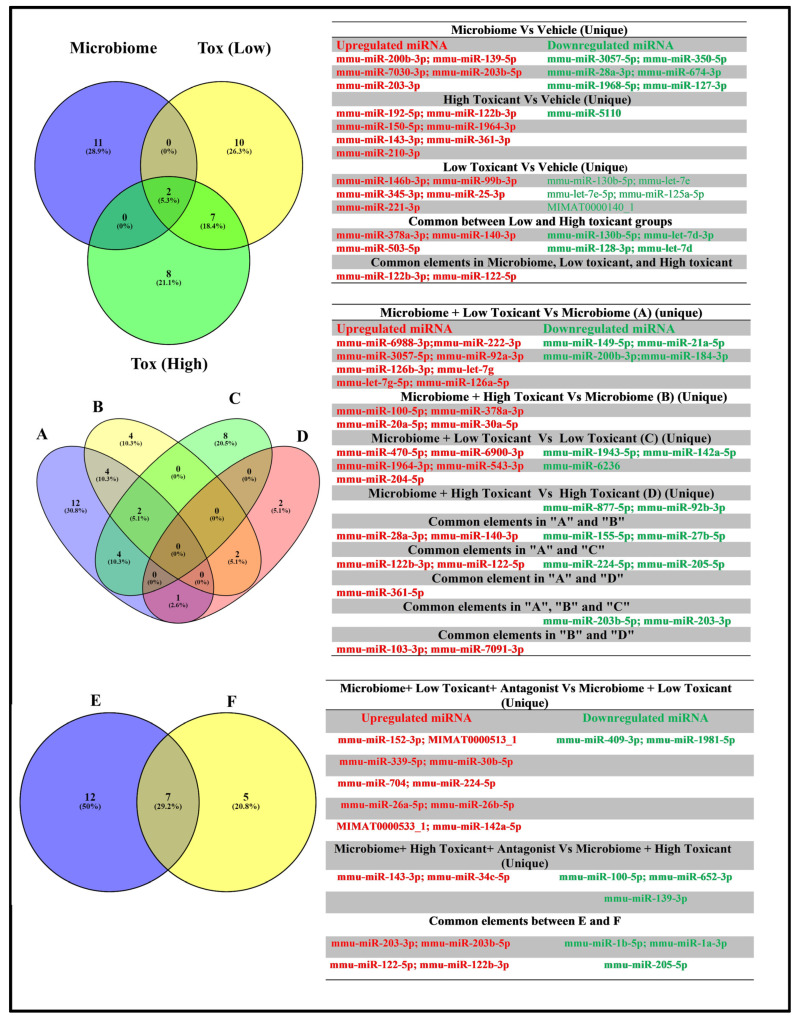
Venn diagram showing differential exo-miRNAs from cultured alveolar macrophages across different treatment groups. **Top** panel: Microbiome-, Low Toxicant-, or High Toxicant-treated alveolar macrophages (AMs) relative to the vehicle-treated AMs. **Middle** panel: Microbiome + Low Toxicant vs. Microbiome (A), Microbiome + High Toxicant vs. Microbiome (B), Microbiome + Low Toxicant vs. Low Toxicant (C), Microbiome + High Toxicant vs. High Toxicant (D). **Bottom** panel: Microbiome+ Low Toxicant+ AHR antagonist vs. Microbiome + Low Toxicant (E), Microbiome+ High Toxicant+ AHR antagonist vs. Microbiome + High Toxicant (F). Numbers in parenthesis in each overlapping segment represent the percent of differential exosomal miRNAs. Names of upregulated and downregulated exo-miRNAs are separately presented as color-coded Lists in the right half of the figure (green = downregulated; red = upregulated). The terms Toxicant and Tox represent B[a}P, with Low (1 µg/mL) and High (10 µg/mL) representing the two concentrations used. The numbers shown within a given segment (unique or overlapping) of a circle in the Venn diagram represent the number of miRNAs belonging to that segment of the circle.

Further analysis using Venn diagrams identified distinct and overlapping exo-miRNAs across different comparisons, with the results summarized in corresponding lists in the figure (Figure 4). When comparing the vehicle-treated group to the microbiome-treated, low toxicant-treated, and high toxicant-treated groups, Venn analysis revealed 11 exo-miRNAs exclusive to the microbiome-treated group, 8 in the high toxicant-treated group, and 10 in the low toxicant-treated group. Seven miRNAs were common between the high and low toxicant-treated groups, while only two exo-miRNAs were common among all three groups (Figure 4, top with list).

Comparisons across the four groups—A (Microbiome + Toxicant_Low vs. Microbiome), B (Microbiome + Toxicant_High vs. Microbiome), C (Microbiome + Toxicant_Low vs. Toxicant_Low), and D (Microbiome + Toxicant_High vs. Toxicant_High)—revealed distinct exo-miRNA distributions. Group A exhibited 12 unique differentially expressed exo-miRNAs, while sEVs from groups B, C, and D contained 4, 8, and 2 exclusive exo-miRNAs, respectively. Overlaps were observed, with four differentially expressed exo-miRNAs common between A and B, four between A and C, one between A and D, and two between B and D. Additionally, two differentially expressed exo-miRNAs were common across A, B, and C (Figure 4, middle with list).

Further comparisons of two additional treatment groups—E (Microbiome + Toxicant_Low + _AHR antagonist vs. Microbiome + _Toxicant_Low) and F (Microbiome + Toxicant_High+ AHR antagonist vs. Microbiome + Toxicant_High)—revealed 12 differentially expressed exo-miRNAs that were unique to E and 5 to F, and 7 differentially exo-miRNAs common between both groups (Figure 4, bottom with list).

### 3.3. Effect of Microbiome Interaction with AMs on the Released exo-miRNA Profile

Comparison of the microbiome-treated alveolar macrophages relative to the vehicle-treated group showed differential expression of 13 significant exo-miRNAs (Supplementary Figure S1). *mmu-miR-7030-p* and *mmu-miR-200b* were highly upregulated with a log_2_-fold-changes of 6.5 and 5.3, respectively, while *mmu-miR350p*, *mmu-miR1965-5p*, and *mmu-miR3057-5p* were highly downregulated with −Log_2_-fold-changes of −6.5, −6.0, and −4.1, respectively.

Enrichment analysis showed that these significant exo-miRNAs (Supplementary Table S2) are involved in regulation of key biological pathways. Specifically, Hippo signaling emerged as the top-regulated pathway. This pathway is primarily involved in regulation of cell proliferation and survival [19,20]. Target gene analysis revealed a total of 353 target genes. In a further analysis of miRNA–target gene interactions, four miRNAs (*mmu-miR-139-5p*; *mmu-miR-122-5p*; *mmu-miR-203-3p*; and *mmu-miR-3057-5p*) regulate four distinct clusters of genes (Supplementary Figure S1). Comparison of low toxicant-treated cells to vehicle-treated alveolar macrophages showed differential expression of 19 significant exo-miRNAs (Supplementary Figure S1), for which enrichment analysis showed ‘Proteoglycans in cancer’ as the top-regulated pathway (Supplementary Table S3). sEVs from high toxicant-treated alveolar macrophages relative to the vehicle-treated group differentially expressed 17 significant exo-miRNAs, with Hippo signaling being the top pathway based on enrichment analysis (Supplementary Figure S1 and Supplementary Table S4).

### 3.4. Effect of Microbiome + Toxicant Interactions with AMs on the Released sEV miRNA Profile

Comparison of the ‘microbiome + low-dose toxicant treated group’ versus the ‘microbiome-only treated group’ showed about 23 significantly differentially expressed exo-microRNAs (Supplementary Figure S2). Five of these microRNAs, namely *mmu-miR6988-3p*, *mmu-mir126b-3p*, *mmu-mir126a-5p*, *mmu-mir3057-5p*, and *mmu-mir-36-5p*, were highly upregulated with log_2_-fold-changes of 7.165, 6.322, 6.322, 3.682, and 2.735, respectively, while *mmu-miR-224-5p*, *mmu-miR-205-5p*, *mmu-miR-149-5p*, *mmu-miR-184-3p*, and *mmu-miR-200b-3p* were highly downregulated with −log_2_-fold-changes of −7.25, −6.39, −3.64, −2.48, and −2.43, respectively. Target gene analysis of these 23 significant exo-miRNAs revealed a total of 966 target genes. Gene target and miRNA interaction analysis demonstrated 14 gene clusters regulated by 14 distinct miRNAs (*mmu-miR-224-5p*, *mmu-mir-205-5p*, *mmu-miR149-5p*, *mmu-miR122-5p*, *mmu-miR-361-5p*, *mmu-miR222-3p*, *mmu-miR-3057-5p*, *mmu-miR200b-3p*, *mmu-miR203-3p*, *mmu-miR92a-3p*, *mmu-miR21a-5p*, *mmu-miR155-5p*, *mmu-miR126a-5p*, and *mmu-let-7g-5p*). Enrichment analysis showed significant KEGG pathways were upregulated, as shown in Supplementary Table S5, with ‘proteoglycans in cancer’ emerging as the top pathway.

Comparison of the ‘microbiome + high-dose toxicant treated group’ versus the ‘microbiome-only treated group’ led to the identification of 12 significantly differentially expressed exo-miRNAs. Eight of these exo-miRNAs were highly upregulated, whereas four were downregulated (Supplementary Figure S2). KEGG pathway analysis highlighted significant enrichment of a number of biological pathways, with the top two pathways related to fatty acid synthesis and metabolism (Supplementary Table S6). Collectively, these significant miRNAs target 252 genes; three of the miRNAs, namely *miR-20a-5p*, *miR-155-5p* and *miR-30a-5p*, controlled three clusters of the target genes (Supplementary Figure S2).

**Figure 5:**
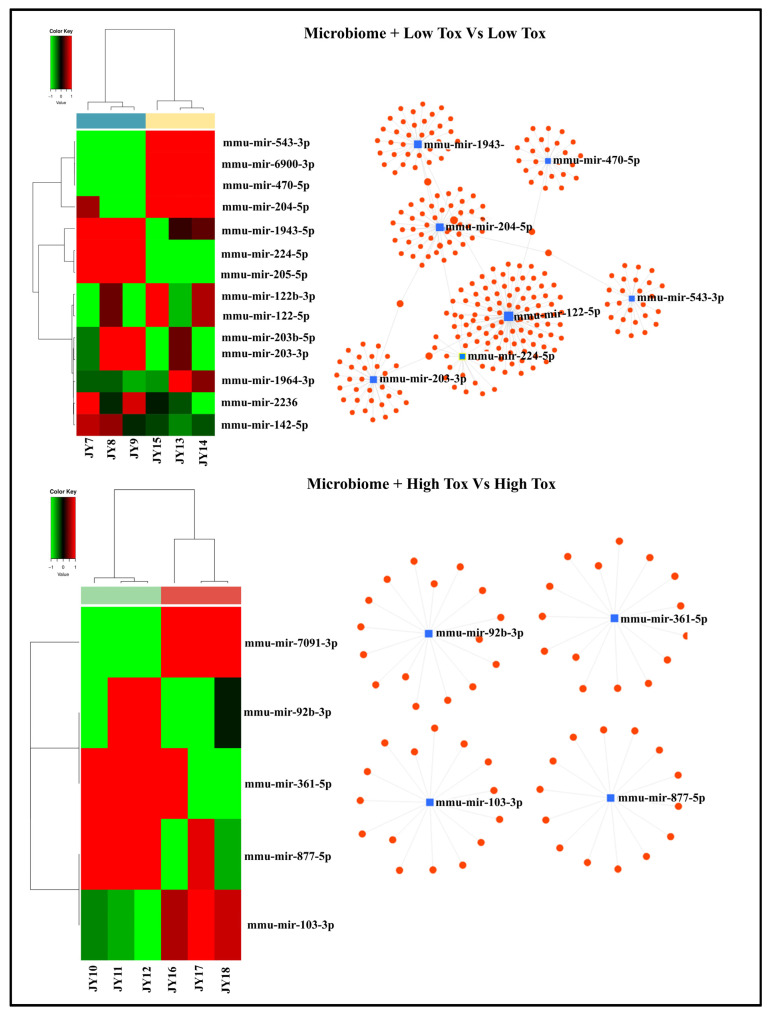
Differential expression of exo-miRNAs and target gene interactions from cultured alveolar macrophages exposed to microbiome and benzo[a]pyrene compared to benzo[a]pyrene only. Left panel: Heat maps of differential exo-miRNA expression. Right panel: exo-miRNA target gene interaction. The top panel is for Microbiome (Mb) + Low Toxicant (Tox) versus Low Tox, and bottom panel is for Mb + High Tox versus High Tox. The term Toxicant or Tox represents B[a}P, with Low (1 µg/mL) and High (10 µg/mL) representing the two concentrations used. Sample ID information (JY1-JY24) is described separately in the Supplementary Materials. Validated targeted genes for corresponding miRNAs are presented separately in the Supplemental Excel Data File.

To assess the impact of the microbiome under low- and high-dose toxicant treatment conditions, we compared the following groups: ‘microbiome + low-dose toxicant’ versus ‘low-dose toxicant only’, and ‘microbiome + high-dose toxicant’ versus ‘high-dose toxicant only’. In both comparisons, significant differentially expressed exo-miRNA were observed. Under low-dose toxicant conditions, 14 exo-miRNAs were differentially expressed by the microbiome-exposed cells, with 7 being highly upregulated. These highly expressed exo-miRNAs (*mmu-miR-1943-5p*, *mmu-miR-470-5p*, *mmu-miR-204-5p*, *mmu-miR-122-5p*, *mmu-miR-543-3p*, *mmu-miR-224-5p*, and *mmu-miR-203-3p*) collectively target approximately 335 genes, forming seven distinct clusters (Figure 5). KEGG pathway analysis identified lysine degradation and Hippo signaling as the top functional categories (Table 1). In contrast, under high-dose toxicant conditions, only five exo-miRNAs were differentially expressed, with just two that were upregulated (*mmu-miR-103-3p* and *mmu-miR-7091-3p*). Four of these exo-miRNAs (*mmu-miR-92b-3p*, *mmu-miR-361-5p*, *mmu-miR-877-5p*, and *mmu-miR-103-3p*) targeted around 64 genes, forming four separate gene clusters (Figure 5). KEGG pathway analysis highlighted fatty acid synthesis, metabolism, and degradation as the predominant pathways (Table 2).

### 3.5. Effect of AHR Antagonist Under Low- and High-Dose PAH Treatment of AMs on exo-miRNA Profile

**Figure 6:**
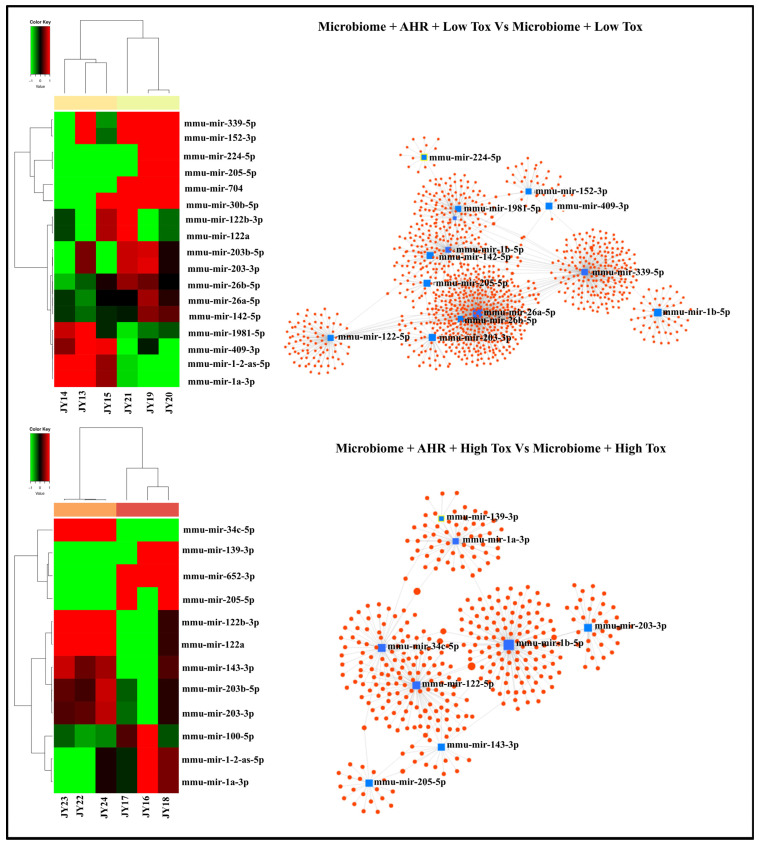
Effect of AHR antagonist on differential exo-miRNA expression and target gene interactions from cultured alveolar macrophages exposed to microbiome, benzo[a]pyrene, and AHR antagonist compared to microbiome and benzo[a]pyrene only. Left panel: Heat maps of differential exo-miRNA expression. Right panel: exo-miRNA target gene interaction. The top panel is for Microbiome + Low Toxicant + AHR antagonist vs. Microbiome + Low Toxicant, and the bottom panel is for Microbiome + High Toxicant + AHR antagonist vs. Microbiome + High Toxicant. The term Toxicant or Tox represents B[a}P, with Low (1 µg/mL) and High (10 µg/mL) representing the two concentrations used. Sample ID information (JY1-JY24) is described separately in the Supplementary Materials. Validated targeted genes for corresponding miRNAs are presented separately in the Supplemental Excel data file.

To check the effect of the AHR antagonist, we compared the microbiome treatment in the presence of low or high doses of the toxicant along with the AHR inhibitor vs. microbiome treatment and toxicant treatment groups only (Figure 4). We found that 12 of the exo-miRNAs were unique to the low-dose conditions, whereas 5 exo-miRNAs were unique to the high-dose conditions, while 7 differentially expressed exo-miRNAs were common between the low-dose and high-dose conditions. Under low-dose toxicant conditions (microbiome + low-dose toxicant treatment + AHR antagonist group) vs. (microbiome+ low-dose toxicant treatment group), we found significant differential expression of 19 exo-miRNAs in terms of log2 fold change, with 15 exo-miRNAs highly upregulated. Target gene analysis showed that 14 of these significant exo-miRNAs targeted about 1259 genes, with 14 different gene clusters (Figure 6). KEGG pathway enrichment analyses are shown in Table 3. Similarly, under high-dose toxicant conditions (microbiome + high-dose toxicant treatment + AHR antagonist group) vs. (microbiome + high-dose toxicant treatment group), there were 12 significantly differentially expressed exo-miRNAs, including 6 exo-miRNAs that were highly expressed. Eight of these 12 significant exo-miRNAs targeted eight gene clusters. Further, KEGG pathway enrichment analysis revealed top pathways such as fatty acid synthesis, proteoglycans in cancer, and the Hippo signaling pathway, as shown in Table 4.

## 4. Discussion

The current study reports the biogenesis/characterization of sEVs and associated miRNA signatures from alveolar macrophages (AMs) as a result of their interactions with lung resident microbiota or when exposed to the ubiquitous toxic PAH B[a]P under different experimental conditions (low versus high dose of B[a]P; with and without lung-extracted microbiota; and with and without an antagonist that blocks the receptor AHR required for PAH uptake). These treatment groups were compared for differential expression of exo-miRNAs. The results clearly demonstrated the release of AM-sEVs carrying differential miRNA cargo, with either strong upregulation or downregulation of critical miRNAs, under different treatment conditions involving microbiota, B[a]P, and/or the AHR antagonist. Several upregulated or downregulated exo-miRNAs were either unique or common across treatment groups, showing the differential modulatory effects of the treatments. Taken together, the data reveal that microbiome treatment diminishes the toxicant effect in alveolar macrophages, exerting a protective role in cancer progression and invasion via regulation of key protective miRNAs.

To further contextualize the biological relevance of the differentially expressed exosomal miRNAs identified in this study, we examined their predicted target genes/proteins, and associated pathways using the published literature and pathway enrichment analyses. Many of the miRNAs regulated by microbiome treatment or B[a]P exposure converge on genes encoding proteins involved in mTOR, FOXO, MAPK, Hippo signaling, lysine degradation, and fatty acid metabolism, all of which play central roles in carcinogenesis, immune regulation, and cellular stress responses. For example, miR-203-3p targets components of the mTOR–FOXO–MAPK axis, which influences longevity, apoptosis, and metabolic adaptation. Several miRNAs altered by high-dose B[a]P exposure (e.g., *miR-210-3p*, *miR-361-3p*, and *miR-150-5p*) are linked to proteins regulating hypoxia responses, mitochondrial metabolism, and tumor progression. Likewise, miRNAs modulated in the microbiome-treated groups map to proteins within the Hippo pathway, a key regulator of cell proliferation and tissue homeostasis and to enzymes involved in lysine catabolism, which supports anti-tumor immunity through histone crotonylation. In the high-toxicant microbiome-treated group, several downregulated miRNAs target genes encoding proteins involved in fatty acid biosynthesis, a metabolic program essential for tumor growth and membrane synthesis. Together, these interactions indicate that the exosomal miRNA cargo released by alveolar macrophages influences downstream protein networks that govern xenobiotic metabolism, inflammation, metabolic reprogramming, and cancer-related signaling, providing mechanistic insight into how the lung microbiome modulates PAH-induced toxicity.

When alveolar macrophages were exposed to the lung microbiome alone, relative to the vehicle control, we observed upregulation of five unique exo-miRNAs: *mmu-miR-200b-3p*, *mmu-miR-139-5p*, *mmu-miR-7030-3p*, *mmu-miR-203b-5p*, and *mmu-miR-203-3p*. In humans, *miR-200b-3p*, a member of the miR-200 family of miRNAs, is found in a cluster on chromosome 1. Expression of these miRNAs seems to be important for regulation of various disease conditions such as cancer and cardiovascular disorders. Expression of *mmu-miR-200b-3p* is significant as miR-200 family miRNAs are involved in the suppression of epithelial to mesenchymal transition (EMT) in certain types of breast cancer. Overexpression of this miRNA has been shown to repress EMT [21]. Similarly, expression of *miR-139-5p* has been shown to suppress EMT in colon cancer. This miRNA exerts its function by targeting the BCL2 pathway to suppress tumor metastasis in colon cancer [22]. Further, *miR-139-5p* has been shown to have a role in the inhibition of breast cancer progression [23], whereas circulating levels of *miR122-5p* are associated with acute myocardial infarction in AMI patients [24]. Similarly, *miR-203-3p* has been shown to regulate esophageal cancer [25]. Another upregulated miRNA, *mmu-miR-7030-3p*, was recently reported to be abundantly found in exosomes secreted by cardiac mesenchymal stem cells, and these exosomes protected acute ischemic myocardium from reperfusion injury [26]. On the other hand, *miR-203b-5p* was found to be downregulated in the sera of colon cancer patients when compared with healthy controls. Upregulation of this miRNA, as observed in our study, might suppress such conditions [27]. Expression of *miR-203-3P*, another upregulated miRNA in our study, was found to be associated with lifespan variations among different mouse strains. Its key target genes in mice are involved in aging and longevity pathways, including mTOR, FOXO, and MAPK. Many of these target genes also show significant links to longevity [28].

The microbiome-treated alveolar macrophages (relative to vehicle control) also showed downregulation of six exo-miRNAs: *mmu-miR-3057-5p*, *mmu-miR-350-5p*, *mmu-miR-28a-3p*, *mmu-miR-674-3p*, *mmu-miR-1968-5p*, and *mmu-miR-127-3p*. Of these, *miR-3057-5p* was found to be upregulated in a Leydig cell line treated with the mycotoxin Zearalenlone (ZEN), an environmental toxicant very harmful to animal and human health via cytotoxic activity. Therefore, downregulation of *mmu-miR-3057-5p* in the microbiome-treated group might confer protection to the cells from cytotoxicity [29]. Another downregulated miRNA, *miR-350*, was found to be upregulated in the liver of a propionic acidemia (PA) mouse model [30]. In the enrichment analysis, the Hippo signaling pathway emerged as the top KEGG pathway. This pathway plays a crucial protective role in cancer by restricting uncontrolled cell proliferation and maintaining tissue homeostasis [31]. Considering the regulation of these miRNAs and their functional relevance in light of the above findings and scientific premise on the impacted miRNAs, the lung microbiome seems to play an important role in protection via regulation of levels of key miRNAs in exosomes.

In the high toxicant (B[a]P) concentration-treated group, we observed upregulation of seven unique miRNAs, namely *mmu-miR-192-5p*, *mmu-miR-122b-3p*, *mmu-miR-150-5p*, *mmu-miR-1964-3p*, *mmu-miR-143-3p*, *mmu-miR-361-3p*, and *mmu-miR-210-3p*, and downregulation of *mmu-miR-5110*, as compared to the vehicle control group. Of these miRNAs, *miR-192-5p* has been associated with oxidative stress in liver acute injury in mice [32] and *miR-122b-3p* with the pathology of lung cancer. Overexpression of *miR-122b-3p* in alveolar epithelial type II cells (A549 cell line) caused inhibition of tumor cell proliferation and induced apoptosis [33]. Expression of *miR-150-5p* has been shown to repress the tumor suppressor gene *TP*53, which in turn promotes colon adenocarcinoma [34]. Overexpression of *miR-361-3p* has been associated with pancreatic duct adenocarcinoma, promoting cancer cell migration and invasion in vitro [35]. Similarly, upregulation of *miR-210-3p*, a well-known oncogenic miRNA, is involved in cancer development, progression, and metastasis [36]. Further, Hippo signaling was the top pathway impacted. Evidence shows that dysregulated Hippo signaling is closely associated with tumorigenesis, invasion, and drug resistance, highlighting the pathway’s importance in preventing malignant transformation [37]. Therefore, as evidenced from the literature, it can be argued that high toxicant (B[a]P) treatment of alveolar macrophages upregulated oncogenic or tumor suppressor miRNAs, which are involved in the promotion of cancer-related diseases.

A low-toxicant (B[a]P)-concentration treatment of alveolar macrophages led to the upregulation of five miRNAs—*mmu-miR-146b-3p*, *mmu-miR-99b-3p*, *mmu-miR-345-3p*, *mmu-miR-25-3p*, and *mmu-miR-221-3p*—and the downregulation of five miRNAs—*mmu-miR-130b-5p*, *mmu-let-7e*, *mmu-let-7e-5p*, *mmu-miR-125a-5p*, and *MIMAT0000140_1* (*mmu-miR-128-3p* variant)—which were unique to this group only. One of these upregulated miRNAs, *miR-99b-3p*, is a potent tumor suppressor, and its ectopic expression has been shown to inhibit oral squamous cell carcinoma cell proliferation [38]. In contrast, *miR-25-3p* and *miR-221-3p* promote tumor growth [39]. *miR-25-3p* promotes the proliferation of triple negative breast cancer by targeting *BTG*2. Among the downregulated miRNAs, *miR-130b-5p* expression has been shown to promote proliferation, migration, and invasion in gastric cancer by targeting *RASAL*1 [40].

Further, ‘proteoglycans in cancer’ emerged as a top-enriched pathway in our analysis, reflecting its central role in coordinating tumor–microenvironment interactions and growth-factor signaling. By regulating extracellular matrix remodeling, cell adhesion, and invasive behavior, proteoglycans actively contribute to cancer progression and help shape a microenvironment that supports tumor survival and dissemination [41]. Collectively, the above published evidence on the roles of the regulated miRNAs suggests that low-toxicant (B[a]P)-concentration treatment could help suppress cancer progression either by upregulating or downregulating the key miRNA expressions. However, some of the miRNAs identified in the low toxicant treatment group are also involved in cancer progression; for example, *miR-125a-5p*, which was downregulated in the current study, is a tumor suppressor in breast cancer [42].

To understand how lung microbiome interacts in the presence of toxicants (as exemplified by the PAH compound B[a]P in this study), we treated the alveolar macrophage cells in the presence of lung-extracted microbiota with either a low toxicant B[a]P dose or a high toxicant B[a]P dose and compared the results with the low- or high-toxicant B[a]P-only treated groups, using a Venn diagram. In the presence of low toxicant concentrations, we found an upregulation of five miRNAs, namely *mmu-miR-470-5p*, *mmu-miR-6900-3p*, *mmu-miR-1964-3p*, *mmu-miR-543-3p*, and *mmu-miR-204-5p*, and downregulation of three miRNAs, namely *mmu-miR-1943-5p*, *mmu-miR-142a-5p*, and *mmu-miR-6236*. Expression of *miR-543-3p* was found to be upregulated in the case of bladder cancer, and its overexpression promoted proliferation and inhibited apoptosis in bladder cancer cells [43]. *miR-204-5p* was found to be downregulated in osteosarcoma and has a tumor suppressor role by targeting *EBF*2, which promotes the migration of osteosarcoma [44]. The role of the other three upregulated miRNAs is currently unknown. Downregulation of *miR-142a-5p* in alveolar macrophages is critical, as a mutant of *miR-142a-5p* in the zebra fish model has been shown to enhance P53 tumor suppressor function and signaling [45]. Further, lysine degradation emerged as the top pathway following enrichment analysis. The lysine degradation pathway plays an emerging role in anti-tumor immunity. Metabolites generated through lysine catabolism enhance histone crotonylation, which promotes transcriptional programs that support T-cell activation and anti-tumor function [46]. This immune-metabolic shift may strengthen tumor surveillance and slow cancer progression, positioning lysine metabolism as a potential therapeutic target. Additionally, dysregulated lysine-derived post-translational modifications, particularly succinylation, have been linked to oncogenic signaling and cancer progression, underscoring the importance of balanced lysine metabolism in tumor biology [47].

The microbiome treatment along with high toxicant concentrations when compared with high toxicant concentration-only treatment, showed no upregulation of any unique miRNA but downregulation of two miRNAs. One of these miRNAs, *mmu-miR-92b-3p*, is essential for the progression of esophageal squamous cell cancer (ESCC), and upregulation of this miRNA promotes ESCC migration and invasion [48]. Enrichment analysis showed the fatty acid metabolism as the top pathway, and three of these four miRNAs (*miR-92b-3p*, *miR-361-5p*, *miR-877-5p*, and *miR-103-3p*) that targeted around 64 genes, forming four separate gene clusters, were downregulated. Fatty acid biosynthesis is a key metabolic program supporting cancer cell proliferation, membrane formation, and oncogenic signaling, and several of the miRNAs examined may influence this lipogenic shift. Among them, *miR-103-3p* shows the strongest metabolic connection, as it suppresses fatty acid oxidation through *ACOX*1 inhibition, thereby increasing intracellular lipid availability that can feed into de novo fatty acid synthesis and support tumor growth [49]. *miR-361-5p*, by targeting *SIRT*1, promotes lipid accumulation in metabolic tissues; because SIRT1 negatively regulates lipogenesis, its suppression may indirectly enhance fatty acid synthesis-related pathways relevant to tumor progression [50]. *miR-877-5p*, implicated in triple-negative breast cancer linked to metabolic syndrome, may contribute to lipogenic reprogramming through *IGF*2-mediated metabolic signaling, a pathway known to activate SREBP1 and FASN in cancer [51]. Although *miR-92b-3p* is primarily associated with proliferation and invasion, its involvement in oncogenic signaling pathways—including Notch and GABRA3 signaling—suggests potential upstream influence on metabolic rewiring, including fatty acid biosynthesis [52]. Together, these miRNAs may modulate the lipid-rich metabolic environment that many tumors rely on, reinforcing the central role of fatty acid synthesis in cancer progression.

We further investigated the effect of the AHR antagonist and microbiome in the presence of either a low toxicant dose or a high toxicant dose and compared them with the high or low toxicant-only groups using a Venn diagram. At high toxicant concentrations of the PAH compound B[a]P, only two unique miRNAs were upregulated, namely *mmu-miR-143-3p* and *mmu-miR-34c-5p*, and three unique miRNAs were downregulated, namely *mmu-miR-100-5p*, *mmu-miR-652-3p*, and *mmu-miR-139-3p*. Expression of *miR-143-3p* has been reported to induce apoptosis and suppress proliferation, migration, and invasion in thyroid cancer cells by targeting the *MSI*2 gene [53]. Expression of *miR-34c-5p* is not only protective in the proliferation of cancer cells but also protects lungs from chronic obstructive pulmonary disease (COPD) by targeting CCl22 [54,55]. *miR-100-5p* targets key genes involved in cancer progression, and its inhibition induces apoptosis in dormant prostate cancer cells [56]. Upregulation of *miR-652-3p* is reported to promote non-small cell lung cancer cell proliferation and metastasis, implying that its downregulation, as observed in our study, might be beneficial in the lungs [57]. However, under these conditions, downregulation of *miR-139-3p* may have an opposite effect, with its expression inhibiting the growth and metastasis of certain types of cancer, such as ovarian cancer [58]. Therefore, the presence of the microbiome and AHR antagonist seems to have a protective role in the onset of carcinogenesis from treatment with high concentrations of the toxicant [BaP].

In comparison, treatment with a low concentration of the toxicant in the presence of the AHR antagonist and microbiome upregulated ten unique miRNAs, namely *mmu-miR-152-3p*, *MIMAT0000513_1*, *mmu-miR-339-5p*, *mmu-miR-30b-5p*, *mmu-miR-704*, *mmu-miR-224-5p, mmu-miR-26a-5p*, *mmu-miR-26b-5p*, *MIMAT0000533_1*, and *mmu-miR-142a-5p*, and downregulated two unique miRNAs, namely *mmu-miR-409-3p* and *mmu-miR-1981-5p*. Of these, *miR-152-3p* is a tumor suppressor and is reported to regulate glioma cell apoptosis and invasion [59]. Upregulation of mmu-*miR-339-5p* in ovarian cancer cells has been shown to inhibit migration and invasion [60]. Expression of *miR-30b-5p*, a tumor suppressor, is involved in the suppression of cell proliferation, metastasis, and EMT in renal carcinoma cells [61]. *miR-224-5p* has been shown to inhibit the proliferation, migration, and invasion in Uveal melanoma by targeting PIK3R3 and AKT3 [62]. Similarly, *miR-26a-5p* and *miR-26b-5p* have also been shown to act as tumor suppressors in hepatocellular carcinoma [63] and in multiple myeloma cells [64]. miR-409-3p, which was downregulated under the chosen treatment conditions, is also a tumor suppressor and suppresses breast cancer cell growth and invasion [65]. The role of the other downregulated miRNA, *miR-1981-5p*, is yet uncharacterized. Collectively, the above pieces of compelling evidence imply that the AHR antagonist in the presence of low toxicant doses and the microbiome mostly promotes anti-cancerous activity, indicating its therapeutic potential in various cancerous conditions.

The findings of this study have important translational implications for understanding and mitigating PAH-induced lung toxicity. By demonstrating that the lung microbiome modulates the exosomal miRNA cargo released by alveolar macrophages during B[a]P exposure, our results identify a previously unrecognized regulatory layer in host–environment interactions. Several of the differentially expressed miRNAs target pathways involved in carcinogenesis, immune regulation, and metabolic reprogramming, suggesting that these exosomal miRNAs may serve as early biomarkers of PAH exposure or predictors of susceptibility to lung injury. Moreover, the observation that microbiome-associated conditions shift miRNA profiles toward protective, anti-tumorigenic pathways highlights the potential for microbiome-based or miRNA-targeted therapeutic strategies to counteract environmental toxicant effects. These insights provide a foundation for future in vivo studies aimed at developing diagnostic tools or interventions that leverage exosomal miRNAs to monitor or modulate PAH-induced respiratory disease.

Although this study provides important insights into how the lung microbiome modulates exosomal miRNA responses to B[a]P exposure, several limitations should be acknowledged. First, the experiments were conducted using an in vitro alveolar macrophage model, which does not fully recapitulate the complexity of the lung microenvironment, including epithelial–immune–microbiome interactions that occur in vivo. Second, while we identified differentially expressed exosomal miRNAs and performed pathway-level interpretation, functional validation of specific miRNA–target gene–protein interactions was beyond the scope of this study and will require targeted mechanistic experiments. Third, the lung-extracted microbiota used here represents a pooled microbial community; therefore, the contribution of individual microbial taxa to miRNA modulation remains unresolved. Finally, although dose-dependent effects of B[a]P were examined, long-term or chronic exposure models were not included, limiting our ability to assess sustained or cumulative toxicant effects. Future in vivo studies integrating single-taxon microbiome manipulations, proteomic validation, and chronic exposure models will comprehensively define the translational relevance of these findings.

## 5. Conclusions

We observed differential miRNA signatures in small extracellular vesicles (sEVs/exosomes) released by alveolar macrophages (AMs) as a result of interaction with resident lung microbiota or/and carcinogenic polycyclic aromatic hydrocarbon B[a]P (at low or high doses). Key exosomal miRNAs known to be involved in the pathology of cancer were found to be either upregulated or downregulated depending on the treatment. While B[a]P treatment induced cancer-promoting miRNAs in AMs, treatment with lung microbiota alone or in combination with a low dose of B[a]P induced tumor suppressor miRNAs known to prevent cancer progression and metastasis and induce apoptosis. Lung microbiota seemed to abrogate the effect of high B[a]P dose by downregulating miRNAs that favored tumorigenesis. The protective effect of the microbiome was further complemented when AM cells were treated with the AHR antagonist, which induced mostly miRNAs that mediate suppression of cell proliferation, invasion, and metastasis. Taken together, this first-of-its-kind study, to our knowledge, demonstrates the crucial interaction of lung microbiota with alveolar macrophages alone or when exposed to environmental toxicant/carcinogen (PAHs, as exemplified by B[a]P) and identifies key miRNAs (known to be involved in the regulation of cancer pathogenesis) in exosomes released by AMs during these exposures. The identified exo-miRNAs could be of critical significance in understanding remote signaling mechanisms within the lung or systemic destinations in PAH exposure models and could serve as novel exposure biomarkers and/or therapeutic targets for intervention in exposed individuals.

## Figures and Tables

**Table 1:** Enrichment for KEGG biological pathways for differentially expressed exo-miRNAs from cultured alveolar macrophages exposed to microbiome and low-dose benzo[a]pyrene compared to low-dose benzo[a]pyrene only.

KEGG Biological Pathways	*p*-Value
Lysine degradation	1.33 × 10^−7^
Hippo signaling pathway	2.17 × 10^−7^
TGF-beta signaling pathway	2.46 × 10^−5^
Estrogen signaling pathway	3.25 × 10^−5^
Biosynthesis of unsaturated fatty acids	0.000173799
Adherens junction	0.000417697
Steroid biosynthesis	0.001276753
Thyroid hormone signaling pathway	0.001855889
Gap junction	0.002890756
Colorectal cancer	0.002890756
FoxO signaling pathway	0.004101321
Pathways in cancer	0.004637989
Arrhythmogenic right ventricular cardiomyopathy (ARVC)	0.00742302
N-Glycan biosynthesis	0.011064668
Signaling pathways regulating pluripotency of stem cells	0.011064668
Notch signaling pathway	0.013139502
Chronic myeloid leukemia	0.013990076
Acute myeloid leukemia	0.016347755
Cell cycle	0.020770759
Prostate cancer	0.021978286
Glycosaminoglycan biosynthesis-heparan sulfate/heparin	0.026334649
Endometrial cancer	0.035505956
Protein processing in endoplasmic reticulum	0.039107493
Degradation of aromatic compounds	0.039644522
Endocytosis	0.039644522

**Table 2:** Enriched KEGG biological pathways for differentially expressed exo-miRNAs from cultured alveolar macrophages exposed to microbiome and high-dose benzo[a]pyrene compared to high-dose benzo[a]pyrene only.

KEGG Biological Pathways	*p*-Value
Fatty acid biosynthesis	2.77 × 10^−22^
Fatty acid metabolism	1.04 × 10^−20^
Fatty acid degradation	1.53 × 10^−9^
FoxO signaling pathway	5.41 × 10^−6^
Lysine degradation	0.000152
Tryptophan metabolism	0.000152
Renal cell carcinoma	0.000152
TGF-beta signaling pathway	0.000327
Pancreatic cancer	0.000487
Adherens junction	0.001344
Huntington’s disease	0.001423
Proteoglycans in cancer	0.00906
MAPK signaling pathway	0.012987
Lysine biosynthesis	0.018029
Inositol phosphate metabolism	0.019501
Bacterial invasion of epithelial cells	0.020233
Chronic myeloid leukemia	0.027713
Thyroid hormone signaling pathway	0.031512
Rap1 signaling pathway	0.031974
Colorectal cancer	0.035589

**Table 3:** Enriched KEGG biological pathways for differentially expressed exo-miRNAs from alveolar macrophages exposed to ‘Microbiome + Low Toxicant+ AHR’ relative to ‘Microbiome +Low Toxicant’.

KEGG Biological Pathways	*p*-Value
Prion diseases	1.39 × 10^−16^
Fatty acid biosynthesis	5.53 × 10^−8^
Proteoglycans in cancer	2.48 × 10^−7^
Adherens junction	7.28 × 10^−6^
FoxO signaling pathway	1.28 × 10^−5^
Hippo signaling pathway	1.72 × 10^−5^
Sphingolipid signaling pathway	2.94 × 10^−5^
Hepatitis B	3.22 × 10^−5^
Fatty acid metabolism	5.29 × 10^−5^
Axon guidance	5.29 × 10^−5^
Oocyte meiosis	0.000133
Renal cell carcinoma	0.000207
Progesterone-mediated oocyte maturation	0.000207
Colorectal cancer	0.000231
Protein processing in endoplasmic reticulum	0.000319
Cell cycle	0.000358
Neurotrophin signaling pathway	0.000358
Pancreatic cancer	0.000445
Prostate cancer	0.000504
ErbB signaling pathway	0.000543
Steroid biosynthesis	0.000826
Dorso-ventral axis formation	0.000826
Estrogen signaling pathway	0.000826
Pathways in cancer	0.001015
Glioma	0.001089
MAPK signaling pathway	0.001484
Acute myeloid leukemia	0.001757
GnRH signaling pathway	0.002497
Endocytosis	0.003176
Phosphatidylinositol signaling system	0.003579
Adrenergic signaling in cardiomyocytes	0.003579
Lysine degradation	0.003758
mTOR signaling pathway	0.003759
Chronic myeloid leukemia	0.004235
Choline metabolism in cancer	0.004235
Regulation of actin cytoskeleton	0.004235
Thyroid cancer	0.004477
Ubiquitin-mediated proteolysis	0.004477
N-Glycan biosynthesis	0.004861
Endometrial cancer	0.005104
Inositol phosphate metabolism	0.006105
Oxytocin signaling pathway	0.006105
Thyroid hormone signaling pathway	0.007054
Chagas disease (American trypanosomiasis)	0.008253
Non-small cell lung cancer	0.009409
Citrate cycle (TCA cycle)	0.012319
TNF signaling pathway	0.012319
T cell receptor signaling pathway	0.012319
TGF-beta signaling pathway	0.012347
p53 signaling pathway	0.014272
Gap junction	0.015336
Glycosaminoglycan biosynthesis-chondroitin sulfate/dermatan sulfate	0.017624
Long-term depression	0.018112
Fatty acid elongation	0.028511
Lysosome	0.034468
Long-term potentiation	0.034863
AMPK signaling pathway	0.03623
Melanogenesis	0.040681
Propanoate metabolism	0.041709
Prolactin signaling pathway	0.042495
Insulin signaling pathway	0.047492
Sphingolipid metabolism	0.047895

**Table 4:** Enriched KEGG biological pathways for differentially expressed exo-miRNAs from cultured alveolar macrophages exposed to microbiome, benzo[a]pyrene, and AHR antagonist compared to microbiome and benzo[a]pyrene only.

KEGG Biological Pathways	*p*-Value
Fatty acid biosynthesis	3.18 × 10^−14^
Proteoglycans in cancer	2.03 × 10^−6^
Thyroid hormone signaling pathway	3.30 × 10^−5^
Hippo signaling pathway	5.20 × 10^−5^
Central carbon metabolism in cancer	0.000694
Chronic myeloid leukemia	0.001287
Fatty acid metabolism	0.00131
FoxO signaling pathway	0.002238
Pathways in cancer	0.002238
Dorso-ventral axis formation	0.002584
Steroid biosynthesis	0.003563
Adherens junction	0.003887
Insulin signaling pathway	0.004742
Hepatitis B	0.0065
Lysine degradation	0.006688
Colorectal cancer	0.007327
ErbB signaling pathway	0.011049
Endometrial cancer	0.014345
Glycosaminoglycan degradation	0.014704
Regulation of actin cytoskeleton	0.014704
Viral carcinogenesis	0.022053
Signaling pathways regulating pluripotency of stem cells	0.023416
Cell cycle	0.0238
Glioma	0.0238
Gap junction	0.028571
GnRH signaling pathway	0.028571
TGF-beta signaling pathway	0.028571
Endocytosis	0.031874
Prostate cancer	0.03482
Bacterial invasion of epithelial cells	0.03482
Pantothenate and CoA biosynthesis	0.035539
Biosynthesis of unsaturated fatty acids	0.036004
Protein processing in endoplasmic reticulum	0.047622

## Data Availability

All the data are presented in the manuscript.

## Supplementary Materials

The following supporting information can be downloaded at https://www.mdpi.com/article/10.3390/cells15080715/s1, Sample ID information: Figure S1: Differential exo-miRNA expression patterns and target gene interactions. Figure S2: Differential exo-miRNA expression and target gene interactions. Table S1: Particle size and concentration of small extracellular vesicles using nanoparticle tracking analysis. Table S2: Enrichment for KEGG biological pathways for miRNAs differentially expressed in small extracellular vesicles secreted from cells exposed to microbiome relative to vehicle. Table S3: Enrichment for KEGG biological pathways for miRNAs differentially expressed in small extracellular vesicles secreted from cells exposed to low toxicant relative to vehicle. Table S4: Enrichment for KEGG biological pathways for miRNAs differentially expressed in small extracellular vesicles secreted from cells exposed to high toxicant relative to vehicle. Table S5: Enrichment for KEGG biological pathways for miRNAs differentially expressed in small extracellular vesicles secreted from cells exposed to microbiome + low toxicant relative to microbiome only. Table S6: Enrichment for KEGG biological pathways for miRNAs differentially expressed in small extracellular vesicles secreted from cells exposed to microbiome + high toxicant relative to microbiome only. Supplementary Excel Data: Target genes.

## Abbreviations

The following abbreviations are used in this manuscript:

B[a]P	Benzo[a]pyrene
PAHs	Polycyclic aromatic hydrocarbons
AMs	Alveolar macrophages
sEVs	Small extracellular vesicles
