# Supplementary material for: Exosomal microRNAs from Alveolar Macrophages Reveal a Protective Role of the Lung Microbiome Against Oncogenic Signaling During PAH Exposure
Source: Cells. 2026 Apr 18;15(8):715. doi: 10.3390/cells15080715 (PMC13114357; doi:10.3390/cells15080715)
Supplement: Supplementary file 1 [file cells-15-00715-s001.zip › Supplementary material_Revised [cells-4255361]_Chandra et al 4.12.26.pdf]

# Supplementary Material

## Sample IDs & treatment groups

| **Treatment group** | **Sample ID** (Exosome RNA for sequencing core) |
|---|---|
| 1) Vehicle-only treated cells | JY1 |
| | JY2 |
| | JY3 |
| 2) Microbiome-only treated cells | JY4 |
| | JY5 |
| | JY6 |
| 3) Toxicant-only treated cells (@ a Low conc of 1 ug/ml); | JY7 |
| | JY8 |
| | JY9 |
| 4) Toxicant-only treated cells (@ a High conc of 10 ug/ml); | JY10 |
| | JY11 |
| | JY12 |
| 5) Microbiome + Toxicant (1 ug/ml) treated | JY13 |
| | JY14 |
| | JY15 |
| 6) Microbiome + Toxicant (10 ug/ml) treated | JY16 |
| | JY17 |
| | JY18 |
| 7) Microbiome + Toxicant (1 ug/ml) in presence of an AHR antagonist | JY19 |
| | JY20 |
| | JY21 |
| 8) Microbiome + Toxicant (10 ug/ml) in presence of an AHR antagonist | JY22 |
| | JY23 |
| | JY24 |
| | |

# Supplementary Figures

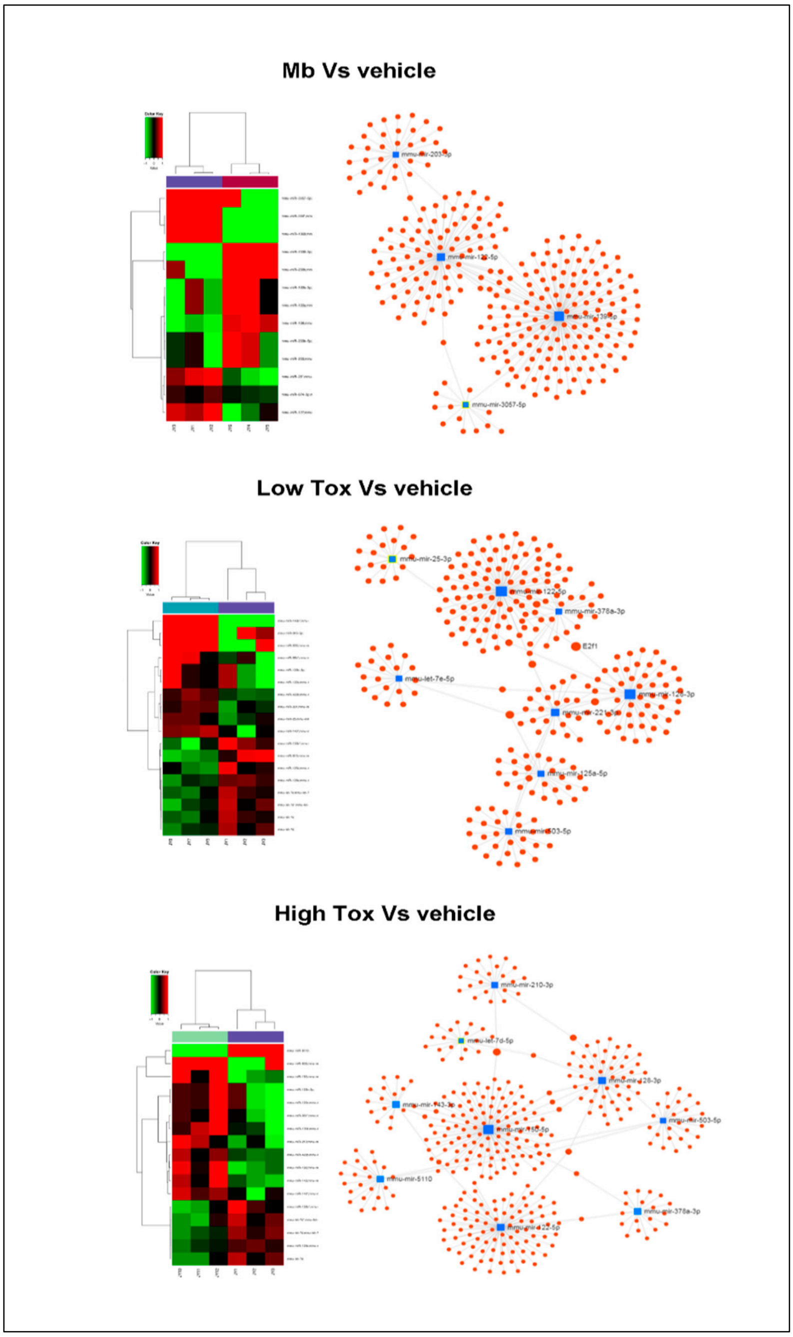


**Supplementary Figure S1: Differential exo-miRNA expression patterns and target gene interactions. Panel Left:** Heat maps; **Panel Right:** miRNA target gene interaction. The **top panel** is for microbiome vs. vehicle control; **middle panel** is low toxicant vs. vehicle control; and **bottom panel** is for high toxicant vs. vehicle control. **Abbreviations**: Mb (Microbiome);Tox (toxicant Benzo[a]pyrene); Low Tox (low dose of the toxicant i.e. 1 μg /ml); High Tox (High dose of the toxicant i.e. 10 μg /ml). The term Toxicant or Tox represents benzo(a)pyrene (B[a}P).

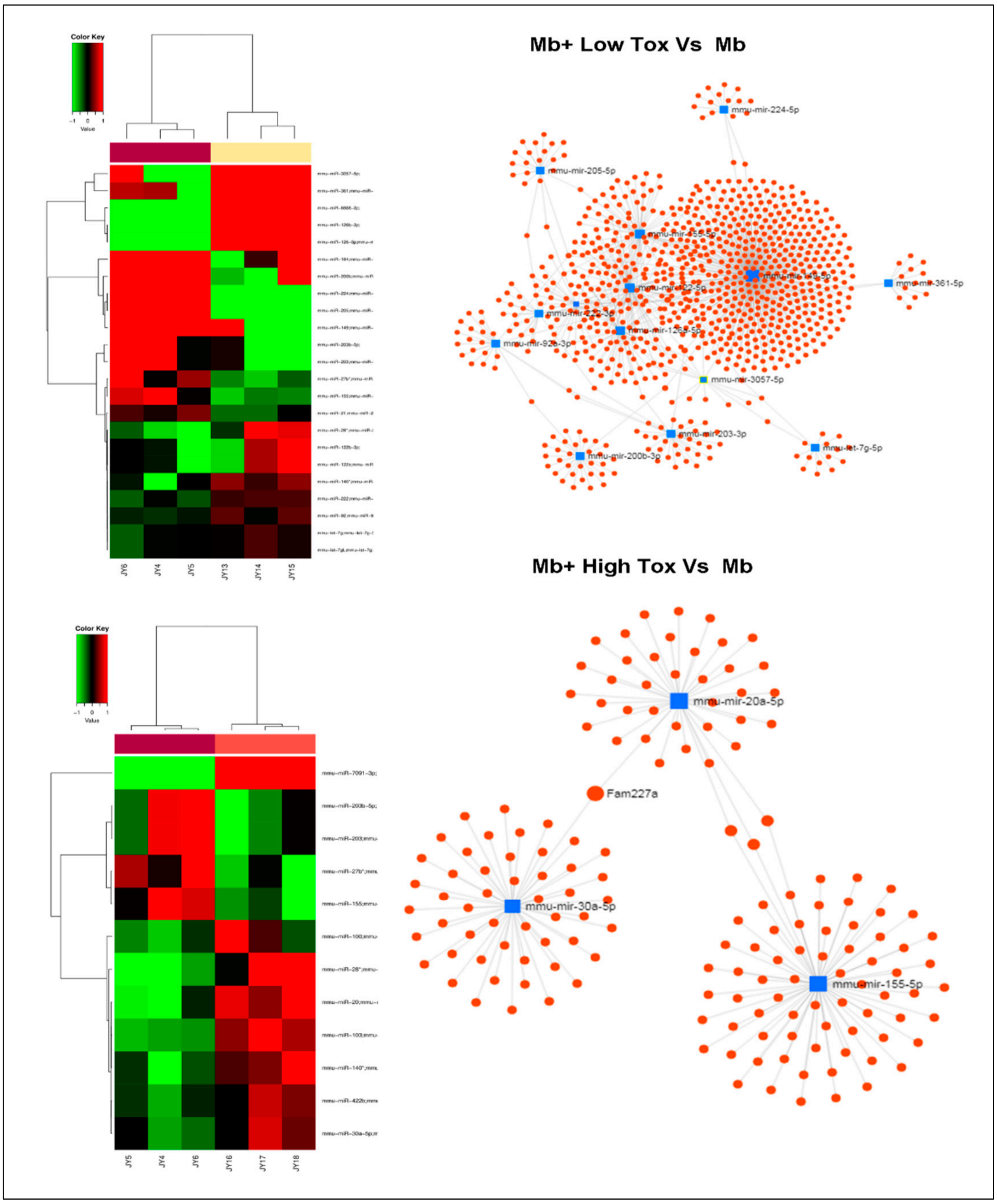


**Supplementary Figure S2: Differential exo-miRNA expression and target gene interactions. Panel Left:** Heat maps and **Panel Right:** miRNA target gene interaction. The **top panel** is for microbiome + low toxicant vs. microbiome; bottom panel is microbiome + high toxicant vs. microbiome. The term Toxicant represents Benzo[a}Pyrene. **Abbreviations**: Mb (Microbiome);Tox (Toxicant), B[a]P (Benzo[a]pyrene); Low Tox (low dose of B[a]P i.e. 1 µg /ml); High Tox (high dose of B[a]P i.e. 10 µg /ml)**.**

# Supplementary tables (S1-S6)

**Supplementary table S1: Particle size and concentration of small extracellular vesicles using nanoparticle tracking analysis**

| Groups* | Sample A | | Sample B | | Sample C | | Sample D | |
|---|---|---|---|---|---|---|---|---|
| | Mean Size **(nm)± SE** | Conc **(Particles/ml)** | Mean Size **(nm)±SE** | Conc **(Particles/ml)** | Mean Size **(nm)±SE** | Conc **(Particles/ml)** | Mean Size **nm)±SE** | Conc **(Particles/ml)** |
| **A** | 146.8 ±10.6 | 2.05e+008 ±1.38e+007 | 134.8±5.1 | 2.99e+008 ±1.87e007 | 157.4±3.8 | 2.28e+008 ±1.99e007 | 144.7±4.6 | 4.23e+008 ±3.7e+007 |
| **B** | 134.5 ±5.0 | 1.21e+008 ±1.58e+007 | 142.6 ±1.4 | 3.90e+008 ±5.69e+007 | 144.5 ±4.9 | 2.67e+008 ±3.74e+007 | 158.3 ±6.4 | 4.30e+008 ±1.85e+007 |
| **C** | 113.8 ±28.8 | 1.29e+008 ±2.09e+007 | 158.1 ±3.3 | 3.18e+008 ±1.98e+007 | 142 ±3.2 | 3.72e+008 ±1.90e+007 | 151.2 ±8.2 | 3.57e+008 ±9.81e+006 |
| **D** | 117.2 ±3.8 | 1.24e+008 ±9.32e+006 | 158.8 ±4.2 | 2.41e+008 ±2.06e+007 | 113.7 ±7.7 | 1.77e+008 ±5.92e+007 | 134.2 ±3.1 | 6.85e+008 ±4.65e+007 |
| **E** | 124.3 ±2.6 | 1.67e+008 ±1.24e+007 | 150.5 ±4.8 | 3.04e+008 ±2.41e+007 | 157.7 ±3 | 3.01e+008 ±1.10e+007 | 143.8 ±3.4 | 3.30e+008 ±2.39e+007 |
| **F** | 122.3 ±5.2 | 1.54e+008 ±2.04e+007 | 153.5 ±6.6 | 2.52e+008 ±1.42e+007 | 117.5 ±6.8 | 1.59e+008 ±1.58e+007 | 126.8 ±2.2 | 5.54e+008 ±3.34e+007 |
| **G** | 100.2 ±44.0 | 8.09e+007 ±9.56e+006 | 95.7 ±24.1 | 1.10e+008 ±1.38e+007 | 151.4 ±7.8 | 2.92e+008 ±1.55e+007 | 155.3 ±1.7 | 3.09e+008 ±3.89e+007 |
| **H** | 139.0 ±7.2 | 2.18e+008 ±1.93e+007 | 130.6 ±2.8 | 4.47e+008 ±3.06e+007 | 132.3 ±11.5 | 1.28e+008 ±1.33e+007 | 142.4 ±5.0 | 2.36e+008 ±6.99e+006 |

***(A)** Vehicle-only treated cultured alveolar macrophage (MHS) cells **(B)** Microbiome-only treated MHS cells **(C)** B[a]P-only treated MHS cells (Low dose: 1 µg/ml) **(D)** B[a]P-only treated MHS cells (high dose: 10 µg/ml) **(E)** Microbiome+B[a]P (1 µg/ml) **(F)** Microbiome+B[a]P (10 µg /ml) **(G)** Microbiome+B[a]P (1 µg /ml) in presence of AHR antagoinst CH223191 (H) Microbiome+B[a]P (10 µg /ml) in presence of AHR antagonist CH223191. Abbreviation: Conc (Concentration).

**Supplementary Table S2: Enrichment for KEGG biological pathways for miRNAs differentially expressed in small extracellular vesicles secreted from cells exposed to microbiome relative to vehicle.**

| KEGG Biological pathways | p-value |
|---|---|
| **Hippo signaling pathway** | 6.64E-05 |
| **TGF-beta signaling pathway** | 0.000479 |
| **Lysine degradation** | 0.001523 |
| **Thyroid hormone signaling pathway** | 0.00228 |
| **Adherens junction** | 0.003041 |
| **Colorectal cancer** | 0.003041 |
| **Pathways in cancer** | 0.0032 |
| **FoxO signaling pathway** | 0.01224 |
| **Porphyrin and chlorophyll metabolism** | 0.016348 |
| **Glycosaminoglycan degradation** | 0.030054 |
| **Sphingolipid metabolism** | 0.035774 |
| **Arachidonic acid metabolism** | 0.044881 |

**Supplementary Table S3: Enrichment for KEGG biological pathways for miRNAs differentially expressed in small extracellular vesicles secreted from cells exposed to low toxicant relative to vehicle.**

| KEGG Biological pathways | p-value |
|---|---|
| **Proteoglycans in cancer** | 2.15E-10 |
| **Thyroid hormone signaling pathway** | 1.49E-09 |
| **Fatty acid biosynthesis** | 6.95E-08 |
| **Hippo signaling pathway** | 1.95E-06 |
| **Pathways in cancer** | 4.59E-06 |
| **Lysine degradation** | 1.51E-05 |
| **Protein processing in endoplasmic reticulum** | 3.23E-05 |
| **Hepatitis B** | 3.23E-05 |
| **Renal cell carcinoma** | 3.82E-05 |
| **mTOR signaling pathway** | 0.000256 |
| **TGF-beta signaling pathway** | 0.000502 |
| **Glycosaminoglycan biosynthesis - chondroitin sulfate / dermatan sulfate** | 0.000739 |
| **FoxO signaling pathway** | 0.000739 |

| | |
|---|---|
| **ErbB signaling pathway** | 0.000739 |
| **MAPK signaling pathway** | 0.000965 |
| **Insulin signaling pathway** | 0.001114 |
| **Fatty acid metabolism** | 0.001541 |
| **Colorectal cancer** | 0.001548 |
| **TNF signaling pathway** | 0.001833 |
| **Acute myeloid leukemia** | 0.003058 |
| **T cell receptor signaling pathway** | 0.003058 |
| **Neurotrophin signaling pathway** | 0.003749 |
| **Estrogen signaling pathway** | 0.005193 |
| **Pancreatic cancer** | 0.005193 |
| **AMPK signaling pathway** | 0.006346 |
| **Chronic myeloid leukemia** | 0.006346 |
| **Transcriptional misregulation in cancer** | 0.006759 |
| **GnRH signaling pathway** | 0.012011 |
| **Wnt signaling pathway** | 0.013255 |
| **HTLV-I infection** | 0.013255 |
| **Adherens junction** | 0.015063 |
| **Prostate cancer** | 0.016617 |
| **Focal adhesion** | 0.017047 |
| **Glioma** | 0.017339 |
| **Fatty acid degradation** | 0.018178 |
| **Valine, leucine and isoleucine degradation** | 0.019889 |
| **PI3K-Akt signaling pathway** | 0.022923 |
| **Dorso-ventral axis formation** | 0.024445 |
| **Citrate cycle (TCA cycle)** | 0.024528 |
| **Small cell lung cancer** | 0.028763 |
| **Selenocompound metabolism** | 0.030162 |
| **Steroid biosynthesis** | 0.030162 |
| **Notch signaling pathway** | 0.030162 |
| **Hepatitis C** | 0.038667 |
| **Axon guidance** | 0.042238 |
| **Chagas disease (American trypanosomiasis)** | 0.042398 |
| **Endocytosis** | 0.042852 |
| **Caffeine metabolism** | 0.043858 |
| **Cell cycle** | 0.044397 |
| **Sulfur relay system** | 0.049363 |

**Supplementary Table S4: Enrichment for KEGG biological pathways for miRNAs differentially expressed in small extracellular vesicles secreted from cells exposed to high toxicant relative to vehicle.**

| KEGG Biological pathways | p-value |
|---|---|

| | |
|---|---|
| **Hippo signaling pathway** | 7.06E-11 |
| **Fatty acid biosynthesis** | 2.27E-09 |
| **Fatty acid metabolism** | 4.69E-07 |
| **Thyroid hormone signaling pathway** | 1.93E-06 |
| **Lysine degradation** | 7.32E-06 |
| **TGF-beta signaling pathway** | 9.25E-06 |
| **Protein processing in endoplasmic reticulum** | 2.88E-05 |
| **Neurotrophin signaling pathway** | 9.22E-05 |
| **Hepatitis B** | 9.22E-05 |
| **mTOR signaling pathway** | 0.000141 |
| **FoxO signaling pathway** | 0.001099 |
| **Valine, leucine and isoleucine degradation** | 0.001387 |
| **Pathways in cancer** | 0.001863 |
| **Proteoglycans in cancer** | 0.00288 |
| **N-Glycan biosynthesis** | 0.006086 |
| **Cell cycle** | 0.006721 |
| **Glioma** | 0.006721 |
| **AMPK signaling pathway** | 0.007383 |
| **Colorectal cancer** | 0.008184 |
| **Central carbon metabolism in cancer** | 0.008184 |
| **Oocyte meiosis** | 0.008184 |
| **Insulin signaling pathway** | 0.009787 |
| **Renal cell carcinoma** | 0.010709 |
| **ErbB signaling pathway** | 0.017258 |
| **Wnt signaling pathway** | 0.019931 |
| **Sulfur relay system** | 0.020411 |
| **Dorso-ventral axis formation** | 0.020411 |
| **Propanoate metabolism** | 0.022241 |
| **Chronic myeloid leukemia** | 0.022241 |
| **Prostate cancer** | 0.022241 |
| **Acute myeloid leukemia** | 0.02291 |
| **Seleno compound metabolism** | 0.027184 |
| **GnRH signaling pathway** | 0.027184 |
| **Endocytosis** | 0.027368 |
| **Pancreatic cancer** | 0.028434 |
| **MAPK signaling pathway** | 0.038142 |
| **T cell receptor signaling pathway** | 0.038142 |
| **HTLV-I infection** | 0.042906 |
| **HIF-1 signaling pathway** | 0.043198 |
| **Notch signaling pathway** | 0.045762 |
| **Signaling pathways regulating pluripotency of stem cells** | 0.045762 |
| **TNF signaling pathway** | 0.046155 |
| **Thyroid cancer** | 0.049905 |

| | |
|---|---|
| **Ubiquitin mediated proteolysis** | 0.049905 |

**Supplementary Table S5: Enrichment for KEGG biological pathways for miRNAs differentially expressed in small extracellular vesicles secreted from cells exposed to microbiome + low toxicant relative to microbiome only .**

| KEGG Biological pathways | p-value |
|---|---|
| **Proteoglycans in cancer** | 3.06E-10 |
| **Renal cell carcinoma** | 6.98E-08 |
| **FoxO signaling pathway** | 2.49E-07 |
| **Lysine degradation** | 7.24E-07 |
| **Glioma** | 7.24E-07 |
| **Hippo signaling pathway** | 7.24E-07 |
| **Thyroid hormone signaling pathway** | 7.24E-07 |
| **Protein processing in endoplasmic reticulum** | 5.71E-06 |
| **Phosphatidylinositol signaling system** | 1.65E-05 |
| **Endocytosis** | 1.65E-05 |
| **Adherens junction** | 1.65E-05 |
| **Chronic myeloid leukemia** | 5.22E-05 |
| **TGF-beta signaling pathway** | 5.45E-05 |
| **ErbB signaling pathway** | 8.21E-05 |
| **Pathways in cancer** | 8.25E-05 |
| **MAPK signaling pathway** | 8.53E-05 |
| **Cell cycle** | 0.000123 |
| **Neurotrophin signaling pathway** | 0.000144 |
| **Estrogen signaling pathway** | 0.000179 |
| **Colorectal cancer** | 0.000179 |
| **Hepatitis B** | 0.000179 |
| **Pancreatic cancer** | 0.000464 |
| **HIF-1 signaling pathway** | 0.000569 |
| **Acute myeloid leukemia** | 0.000686 |
| **Insulin signaling pathway** | 0.001129 |
| **Galactose metabolism** | 0.001184 |
| **Prostate cancer** | 0.001184 |
| **Regulation of actin cytoskeleton** | 0.00145 |
| **Focal adhesion** | 0.001578 |
| **AMPK signaling pathway** | 0.002307 |
| **Central carbon metabolism in cancer** | 0.002758 |
| **GnRH signaling pathway** | 0.002876 |

| | |
|---|---|
| **mTOR signaling pathway** | 0.003602 |
| **Inositol phosphate metabolism** | 0.003649 |
| **PI3K-Akt signaling pathway** | 0.003649 |
| **Steroid biosynthesis** | 0.003839 |
| **Small cell lung cancer** | 0.003975 |
| **Axon guidance** | 0.004098 |
| **Ubiquitin mediated proteolysis** | 0.004986 |
| **Circadian rhythm** | 0.005441 |
| **Bacterial invasion of epithelial cells** | 0.013261 |
| **Biosynthesis of unsaturated fatty acids** | 0.014191 |
| **Fc gamma R-mediated phagocytosis** | 0.015414 |
| **Valine, leucine and isoleucine degradation** | 0.018744 |
| **T cell receptor signaling pathway** | 0.018744 |
| **Non-small cell lung cancer** | 0.020693 |
| **Wnt signaling pathway** | 0.026387 |
| **Propanoate metabolism** | 0.032787 |
| **Prolactin signaling pathway** | 0.038696 |
| **HTLV-I infection** | 0.039519 |
| **Lysosome** | 0.039531 |
| **Endometrial cancer** | 0.044491 |

**Supplementary Table S6: Enrichment for KEGG biological pathways for miRNAs differentially expressed in small extracellular vesicles secreted from cells exposed to microbiome + high toxicant relative to microbiome only.**

| KEGG Biological pathways | p-value |
|---|---|
| **Fatty acid biosynthesis** | 2.15E-09 |
| **Fatty acid metabolism** | 8.04E-08 |
| **Lysine degradation** | 5.41E-07 |
| **Hippo signaling pathway** | 8.70E-07 |
| **Colorectal cancer** | 8.70E-07 |
| **Renal cell carcinoma** | 8.70E-07 |
| **Adherens junction** | 7.03E-06 |
| **Cell cycle** | 7.64E-06 |
| **Endocytosis** | 1.65E-05 |
| **Axon guidance** | 2.66E-05 |
| **Thyroid hormone signaling pathway** | 2.70E-05 |
| **Fatty acid degradation** | 3.52E-05 |
| **N-Glycan biosynthesis** | 4.44E-05 |
| **Protein processing in endoplasmic reticulum** | 4.44E-05 |
| **Pathways in cancer** | 4.44E-05 |

| | |
|---|---|
| **Pancreatic cancer** | 6.37E-05 |
| **TGF-beta signaling pathway** | 9.15E-05 |
| **Hepatitis B** | 9.15E-05 |
| **FoxO signaling pathway** | 0.000665 |
| **Glioma** | 0.000687 |
| **Proteoglycans in cancer** | 0.001214 |
| **MAPK signaling pathway** | 0.001731 |
| **T cell receptor signaling pathway** | 0.002157 |
| **HTLV-I infection** | 0.005786 |
| **Prostate cancer** | 0.00774 |
| **Ubiquitin mediated proteolysis** | 0.009166 |
| **HIF-1 signaling pathway** | 0.010925 |
| **Osteoclast differentiation** | 0.015447 |
| **Non-small cell lung cancer** | 0.015497 |
| **Chronic myeloid leukemia** | 0.015497 |
| **mTOR signaling pathway** | 0.015681 |
| **Acute myeloid leukemia** | 0.015681 |
| **Inflammatory bowel disease (IBD)** | 0.021319 |
| **Glycosaminoglycan biosynthesis - heparan sulfate / heparin** | 0.02282 |
| **Insulin signaling pathway** | 0.02282 |
| **Inositol phosphate metabolism** | 0.025994 |
| **Pantothenate and CoA biosynthesis** | 0.026558 |
| **ErbB signaling pathway** | 0.028321 |
| **Tryptophan metabolism** | 0.032712 |
| **p53 signaling pathway** | 0.032712 |
| **Endometrial cancer** | 0.032712 |
| **Wnt signaling pathway** | 0.033855 |
| **Neurotrophin signaling pathway** | 0.033855 |
| **Ras signaling pathway** | 0.034202 |
| **Arrhythmogenic right ventricular cardiomyopathy (ARVC)** | 0.038928 |
| **Vitamin B6 metabolism** | 0.041056 |
| **Viral carcinogenesis** | 0.045305 |
